# Supplementary figures and images for: Complete chloroplast genomes of two Siraitia Merrill species: Comparative analysis, positive selection and novel molecular marker development
Source: PLoS One. 2019 Dec 20;14(12):e0226865. doi: 10.1371/journal.pone.0226865 (PMC6924677; doi:10.1371/journal.pone.0226865)

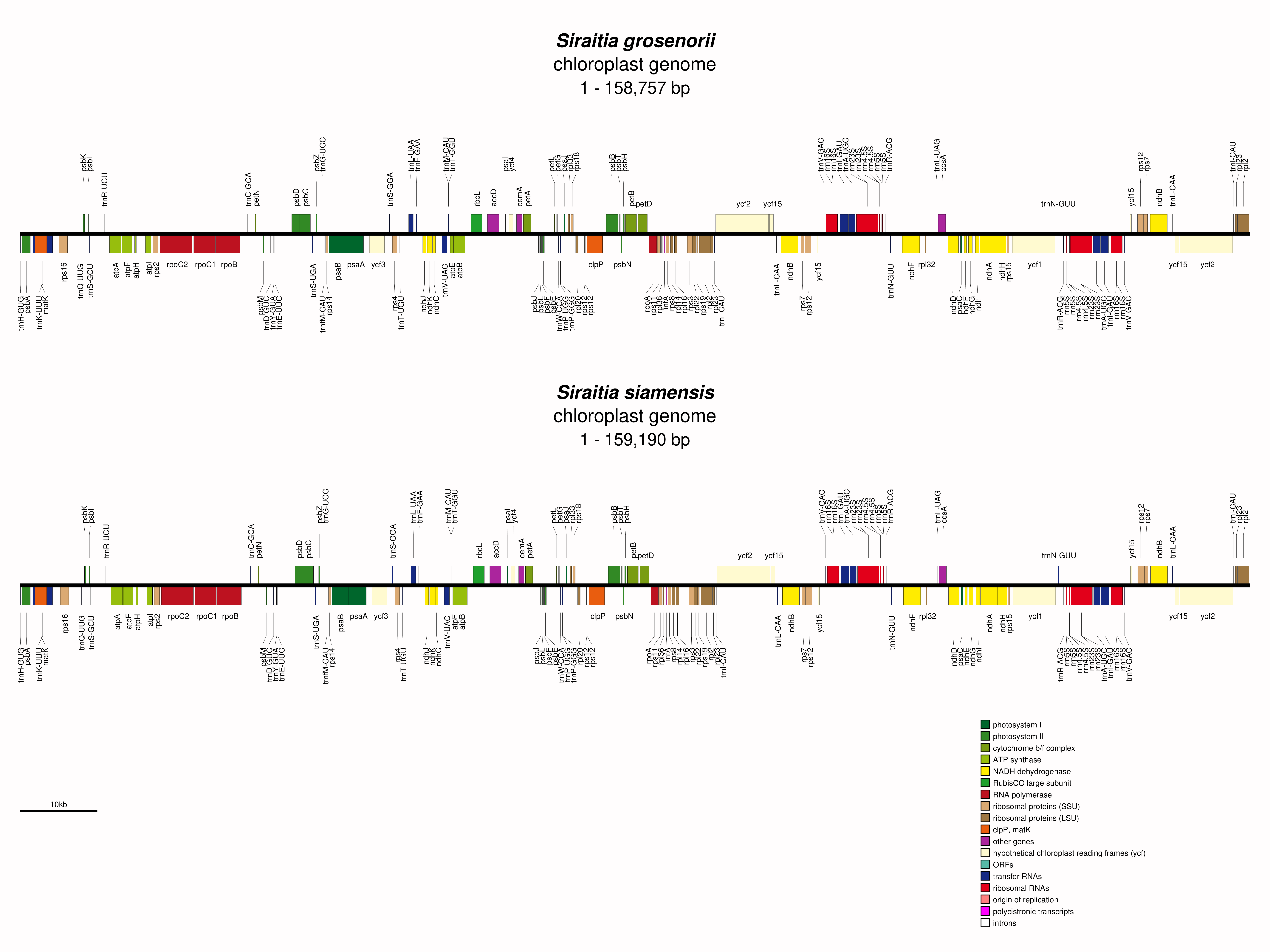

Supplement: S1 Fig — (TIF) [file pone.0226865.s001.tif]

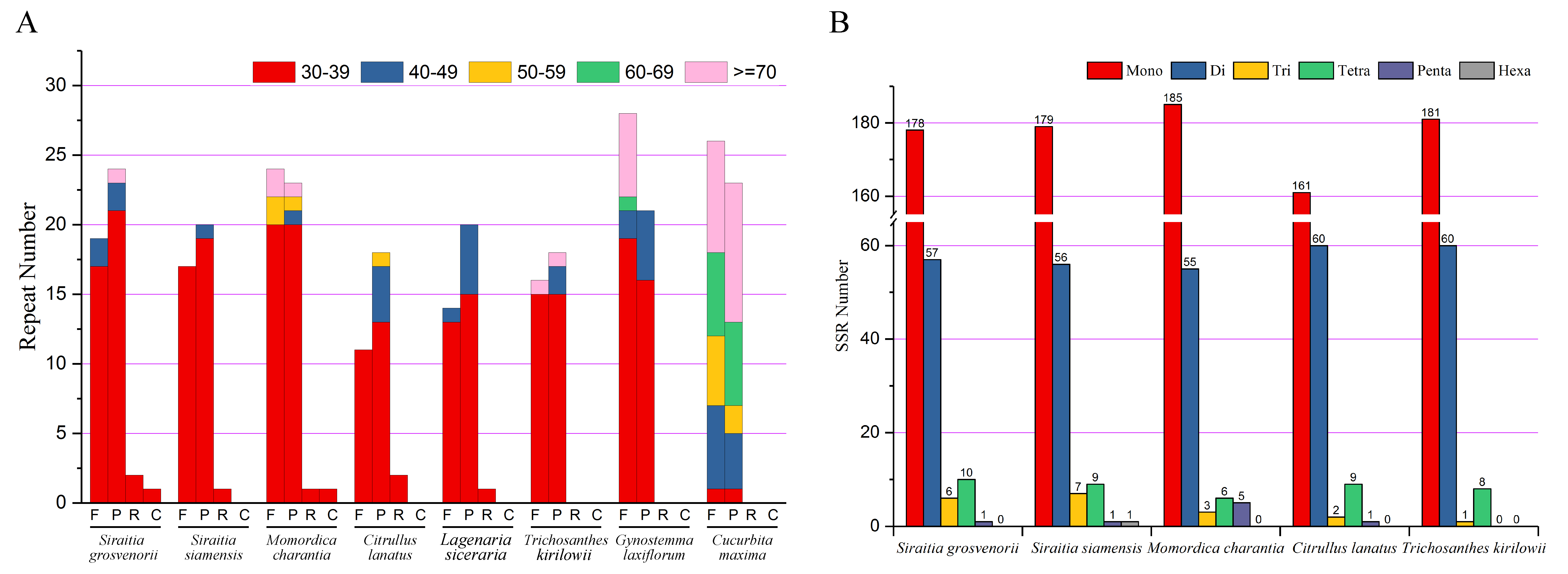

Supplement: S2 Fig — (A) Repeat sequence in eight chloroplast genomes. Repeat sequences were identified by REPuter with length ≥30bp and sequence identified ≥90%. F, P, R, and C are the abbreviation of repeat type F (forward), P (palindrome), R (reverse) and C (complement), respectively. Different length repeat sequences are colored correspondingly. (B) Analysis of simple sequence repeat (SSRs) in chloroplast genomes of five species. (TIF) [file pone.0226865.s002.tif]

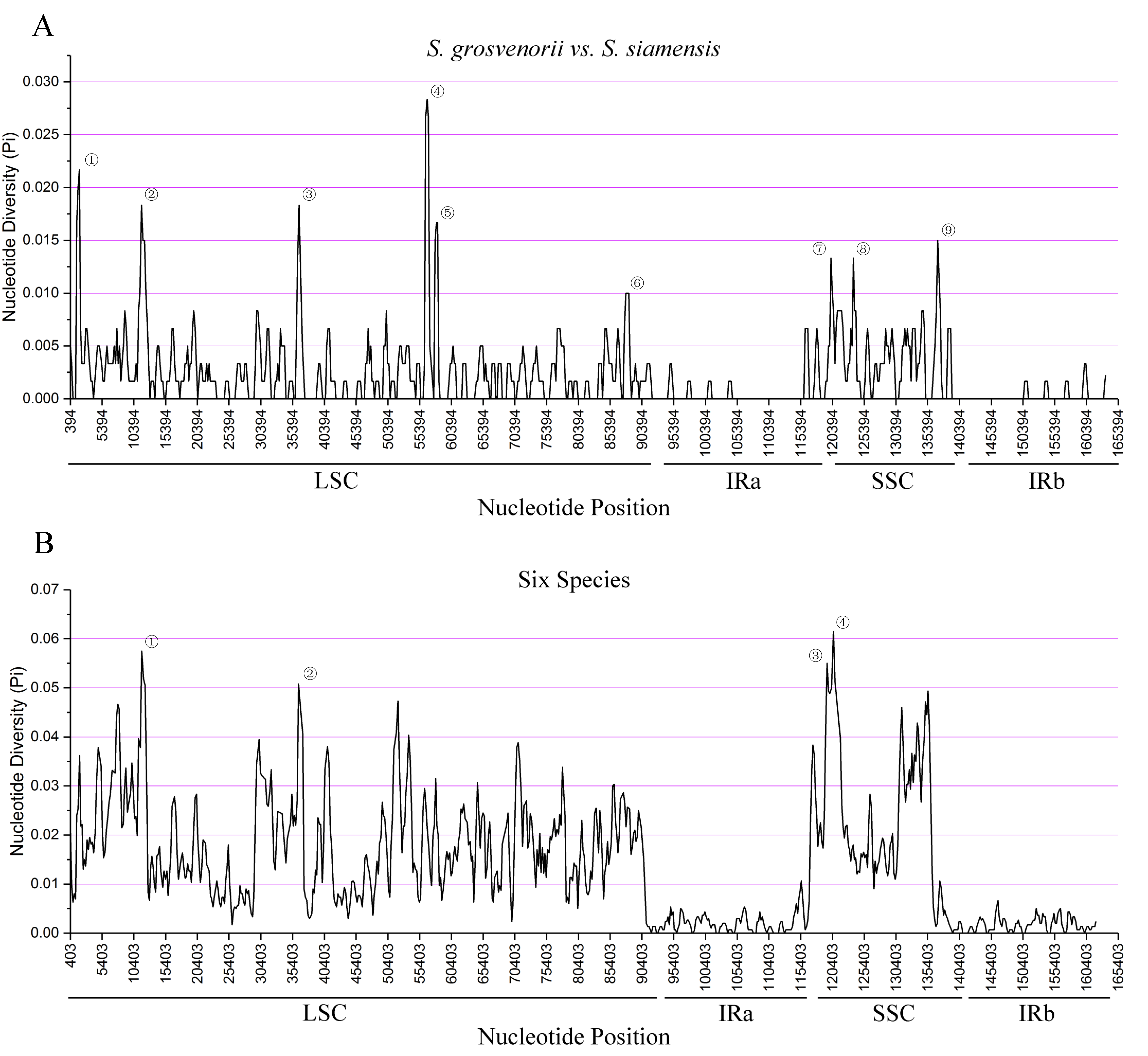

Supplement: S3 Fig — Window length: 600 sites, Step size: 200 sites. X-axis: position of the midpoint of a window; Y-axis: nucleotide diversity (π) of each window. (A) Pi among S. grosvenorii and S. siamensis; (B) Pi among six species of Cucurbitaceae. (TIF) [file pone.0226865.s003.tif]
