# Supplementary material for: Complete chloroplast genomes of two Siraitia Merrill species: Comparative analysis, positive selection and novel molecular marker development
Source: PLoS One. 2019 Dec 20;14(12):e0226865. doi: 10.1371/journal.pone.0226865 (PMC6924677; doi:10.1371/journal.pone.0226865)
Supplement: S1 Table — (DOCX) [file pone.0226865.s004.docx]

**S1 Table. Primer sequence at the boundaries between single cope and IR regions.**

| **Species** | **Regions** | **Forward/Reverse** | **Primer sequence (5’ to 3’)** |
| --- | --- | --- | --- |
| *Siraitia. grosvenorii* | LSC-IRa | F | GGTAGATGCTCGGGACCAAGTT |
|  |  | R | ATAAGCCAGATGAAGGAACGGG |
|  | IRa-SSC | F | GGGAAAGCGAGGAAGAAACAGA |
|  |  | R | GTATAGGGTCTTATCGGGCTGC |
|  | SSC-IRb | F | TGGATTCGTCTGCGGTAAGGTC |
|  |  | R | GGACTAAACAGGAACAAGAGGG |
|  | IRb-LSC | F | CATCTACATCCAGAAAGCCG |
|  |  | R | GCTCCCTATTCAGTGCTATG |
| *Siraitia siamensis* | LSC-IRa | F | GTATAATGGTAGATGCTCGGGC |
|  |  | R | CATAAGCCAGATGAAGGAACGG |
|  | IRa-SSC | F | GGGAAAGCGAGGAAGAAACAGA |
|  |  | R | CTACGATGGTAGCAGCGGGAAT |
|  | SSC-IRb | F | ATGGATTCGTCTGCGGTAAGGT |
|  |  | R | GGACTAAACAGGAACAAGAGGG |
|  | IRb-LSC | F | TCAGCAACAGTCGGACAAGTGG |
|  |  | R | ATTCCAGGCTGAGCACAACATC |
