# Supplementary material for: Complete chloroplast genomes of two Siraitia Merrill species: Comparative analysis, positive selection and novel molecular marker development
Source: PLoS One. 2019 Dec 20;14(12):e0226865. doi: 10.1371/journal.pone.0226865 (PMC6924677; doi:10.1371/journal.pone.0226865)
Supplement: S2 Table — (DOCX) [file pone.0226865.s005.docx]

**S2 Table. List of chloroplast genome sequences used in the study.**

| **Species Name** | **Genome type** | **GenBanK Accession** | **Length (bp)** |
| --- | --- | --- | --- |
| *Cucurbita maxima* | chloroplast | MF991115.1 | 157,204 |
| *Cucurbita moschata* | chloroplast | MF991116.1 | 157,644 |
| *Cucurbita pepo* | chloroplast | MH031787.1 | 157,343 |
| *Cucumis sativus* | chloroplast | AJ970307.1 | 155,293 |
| *Cucumis melo subsp. Melo* | chloroplast | JF412791.1 | 156,017 |
| *Cucumis melo var. flexuosus* | chloroplast | MF536707.1 | 155,815 |
| *Citrullus amarus* | chloroplast | MF536694.1 | 157,008 |
| *Citrullus colocynthis* | chloroplast | MF357889.1 | 157,147 |
| *Citrullus lanatus* | chloroplast | KY014105.1 | 156,906 |
| *Citrullus rehmii* | chloroplast | MF536695.1 | 157,135 |
| *Lagenaria siceraria* | chloroplast | MG022623.1 | 157,145 |
| *Coccinia grandis* | chloroplast | KX147311.1 | 157,035 |
| *Gomphogyne cissiformis var. cissiformis* | chloroplast | MH256801.1 | 157,334 |
| *Gomphogyne cissiformis var. villosa* | chloroplast | MF784515.1 | 156,585 |
| *Gynostemma cardiospermum* | chloroplast | KX852299.1 | 159,042 |
| *Gynostemma caulopterum* | chloroplast | MF136487.1 | 157,937 |
| *Gynostemma compressum* | chloroplast | KY817143.1 | 157,960 |
| *Gynostemma laxiflorum* | chloroplast | MF136486.1 | 158,273 |
| *Gynostemma longipes* | chloroplast | MF152730.1 | 157,601 |
| *Gynostemma pentagynum* | chloroplast | KY670737.1 | 157,791 |
| *Gynostemma pentaphyllum* | chloroplast | KX852298.1 | 158,004 |
| *Gynostemma pubescens* | chloroplast | MF152732.1 | 157,666 |
| *Hemsleya lijiangensis* | chloroplast | MG733988.1 | 158,275 |
| *Hodgsonia macrocarpa* | chloroplast | NC_039628.1 | 156,146 |
| *Trichosanthes kirilowii* | chloroplast | MK036046.1 | 157,481 |
| *Momordica charantia* | chloroplast | MG022622.1 | 158,844 |
| *Arabidopsis thaliana* | chloroplast | NC_000932.1 | 154,478 |
| *Nicotiana tabacum* | chloroplast | NC_001879.2 | 155,943 |
