# Supplementary material for: Complete chloroplast genomes of two Siraitia Merrill species: Comparative analysis, positive selection and novel molecular marker development
Source: PLoS One. 2019 Dec 20;14(12):e0226865. doi: 10.1371/journal.pone.0226865 (PMC6924677; doi:10.1371/journal.pone.0226865)
Supplement: S3 Table — (DOCX) [file pone.0226865.s006.docx]

**S3 Table. Base composition in the chloroplast genomes of *S. grosvenorii* and *S. siamensis*.**

| **Species** | **Region** | **Position** | **T(U) (%)** | **C (%)** | **A (%)** | **G (%)** | **Length (bp)** |
| --- | --- | --- | --- | --- | --- | --- | --- |
| *S. grosvenorii* | LSC | - | 33.5 | 17.7 | 31.9 | 16.9 | 87,625 |
|  | SSC | - | 34.3 | 16.0 | 34.6 | 14.5 | 18,656 |
|  | IRa | - | 28.5 | 20.6 | 28.7 | 22.2 | 26,288 |
|  | IRb | - | 28.7 | 22.2 | 28.5 | 20.6 | 26,288 |
|  | Total | - | 32.0 | 18.7 | 31.2 | 18.1 | 158,757 |
|  | CDS | - | 31.4 | 17.7 | 30.8 | 20.1 | 79,527 |
|  | - | 1st position | 23.8 | 18.7 | 30.6 | 26.9 | 26,509 |
|  | - | 2nd positon | 32.5 | 20.2 | 29.5 | 17.8 | 26,509 |
|  | - | 3rd position | 38.0 | 14.1 | 32.2 | 15.7 | 26,509 |
|  | - | rRNA | 18.8 | 23.6 | 25.8 | 31.7 | 9,050 |
|  | - | tRNA | 25.3 | 23.6 | 21.7 | 29.4 | 2,772 |
| *S. siamensis* | LSC | - | 33.5 | 17.7 | 31.9 | 16.9 | 88,069 |
|  | SSC | - | 34.4 | 16.1 | 34.9 | 14.6 | 18,543 |
|  | IRa | - | 28.5 | 20.6 | 28.7 | 22.2 | 26,289 |
|  | IRb | - | 28.7 | 22.2 | 28.5 | 20.6 | 26,289 |
|  | Total | - | 32.0 | 18.7 | 31.2 | 18.1 | 159,190 |
|  | CDS | - | 31.4 | 17.7 | 30.7 | 20.2 | 79,524 |
|  | - | 1st position | 23.8 | 18.7 | 30.6 | 26.9 | 26,508 |
|  | - | 2nd positon | 32.5 | 20.2 | 29.5 | 17.8 | 26,508 |
|  | - | 3rd position | 38.0 | 14.1 | 32.1 | 15.8 | 26,508 |
|  | - | rRNA | 18.8 | 23.6 | 25.8 | 31.7 | 9,050 |
|  | - | tRNA | 25.3 | 23.6 | 21.7 | 29.4 | 2,772 |
