# Supplementary material for: Complete chloroplast genomes of two Siraitia Merrill species: Comparative analysis, positive selection and novel molecular marker development
Source: PLoS One. 2019 Dec 20;14(12):e0226865. doi: 10.1371/journal.pone.0226865 (PMC6924677; doi:10.1371/journal.pone.0226865)
Supplement: S4 Table — (DOCX) [file pone.0226865.s007.docx]

**S4 Table. Genes contained in the chloroplast genomes of *S. grosvenorii* and *S. siamensis*.**

| **Gene Category** | **Gene Group** | **Gene name** | **Amount** |
| --- | --- | --- | --- |
| Self replication | rRNA genes | *rrn16*(×2), *rrn23*(×2), *rrn4.5*(×2), *rrn5*(×2) | 8 |
|  | tRNA genes | *trnC-GCA*, *trnD-GUC*, *trnE-UUC*, *trnF-GAA*, *trnfM-CAU*, *trnG-UCC*, *trnH-GUG*, *trnK-UUU**, *trnL-UAA**, *trnL-UAG*, *trnM-CAU*, *trnP-GGG*, *trnP-UGG*, *trnQ-UUG*, *trnR-UCU*, *trnS-GCU*, *trnS-GGA*, *trnS-UGA*, *trnT-GGU*, *trnT-UGU*, *trnV-UAC**, *trnW-CCA*, *trnY-GUA*, *trnA-UGC**(×2), *trnI-GAU**(×2), *trnI-UAU*(×2), *trnL-CAA*(×2), *trnN-GUU*(×2), *trnR-ACG*(×2), *trnV-GAC*(×2) | 37 |
|  | Large subunit of ribosome | *rpl2**(×2), *rpl14*, *rpl16*, *rpl20*, *rpl22*, *rpl23*(×2), *rpl32*, *rpl33*, *rpl36* | 11 |
|  | Small subunit of ribosome | *rps2*, *rps3*, *rps4*, *rps7*(×2), *rps8*, *rps11*, *rps12**(×2), *rps14*, *rps15*, *rps16**, *rps18*, *rps19* | 14 |
|  | DNA dependent RNA polymerase | *rpoA*, *rpoB*, *rpoC1**, *rpoC2* | 4 |
| Genes for photosynthesis | Subunits of NADH-dehydrogenase | *ndhA**, *ndhB**(×2), *ndhC*, *ndhD*, *ndhE*, *ndhF*, *ndhG*, *ndhH*, *ndhI*, *ndhJ*, *ndhK* | 12 |
|  | Subunits of photosystem I | *psaA*, *psaB*, *psaC*, *psaI*, *psaJ* | 5 |
|  | Subunits of photosystem II | *psbA*, *psbB*, *psbC*, *psbD*, *psbE*, *psbF*, *psbH*, *psbI*, *psbJ*, *psbK*, *psbL*, *psbM*, *psbN*, *psbT*, *psbZ* | 15 |
|  | Subunits of cytochrome b/f complex | *petA*, *petB**, *petD**, *petG*, *petL*, *petN* | 6 |
|  | Subunits of ATP synthase | *atpA*, *atpB*, *atpE*, *atpF**, *atpH*, *atpI* | 6 |
|  | Large subunit of rubisco | *rbcL* | 1 |
| Other genes | Translational initiation factor | *infA* | 1 |
|  | Maturase | *matK* | 1 |
|  | Protease | *clpP*** | 1 |
|  | Envelope membrane protein | *cemA* | 1 |
|  | Subunit of Acetyl-CoA-carboxylase | *accD* | 1 |
|  | c-type cytochrom synthesis gene | *ccsA* | 1 |
| Genes of unknown functions, Open Reading Frames (ORF, ycf) | | *ycf1*, *ycf2*(×2), *ycf3***, *ycf4*, *ycf15*(×2), *ycf15-ORF**(×2) | 9 |

* Gene contains one intron; ** gene contains two introns; (×2) indicates the number of the repeat unit is two.
