# Supplementary material for: Complete chloroplast genomes of two Siraitia Merrill species: Comparative analysis, positive selection and novel molecular marker development
Source: PLoS One. 2019 Dec 20;14(12):e0226865. doi: 10.1371/journal.pone.0226865 (PMC6924677; doi:10.1371/journal.pone.0226865)
Supplement: S5 Table — (DOCX) [file pone.0226865.s008.docx]

**S5 Table. Location information of genes with introns in the chloroplast genome of *S. grosvenorii* and *S. siamensis*.**

| **Species** | **Gene** | **Location** | **Strand** | **Start** | **End** | **Exon I (bp)** | **Intron I (bp)** | **Exon II (bp)** | **Intron II (bp)** | **Exon III (bp)** |
| --- | --- | --- | --- | --- | --- | --- | --- | --- | --- | --- |
| *S. grosvenorii* | *trnK-UUU* | LSC | - | 1631 | 4200 | 35 | 2498 | 37 |  |  |
|  | *rps16* | LSC | - | 5025 | 6138 | 48 | 853 | 213 |  |  |
|  | *atpF* | LSC | - | 13122 | 14435 | 150 | 756 | 408 |  |  |
|  | *rpoC1* | LSC | - | 22410 | 25194 | 435 | 739 | 1611 |  |  |
|  | *ycf3* | LSC | - | 45088 | 47065 | 126 | 729 | 228 | 742 | 153 |
|  | *trnL-UAA* | LSC | + | 50161 | 50774 | 35 | 529 | 50 |  |  |
|  | *trnV-UAC* | LSC | - | 54439 | 55123 | 37 | 610 | 38 |  |  |
|  | *clpP* | LSC | - | 73173 | 75217 | 69 | 834 | 297 | 620 | 225 |
|  | *petB* | LSC | + | 78157 | 79590 | 6 | 786 | 642 |  |  |
|  | *petD* | LSC | + | 79783 | 80994 | 8 | 729 | 475 |  |  |
|  | *rpl2* | IRa | - | 87690 | 89179 | 393 | 626 | 471 |  |  |
|  | *ycf15-orf* | IRa | + | 96742 | 97274 | 90 | 284 | 159 |  |  |
|  | *ndhB* | IRa | - | 98247 | 100465 | 777 | 686 | 756 |  |  |
|  | *rps12-5' end* | IRa | - | 101315 | 102112 | 232 | 540 | 26 |  |  |
|  | *trnI-GAU* | IRa | + | 105850 | 106879 | 42 | 958 | 30 |  |  |
|  | *trnA-UGC* | IRa | + | 106952 | 107826 | 38 | 802 | 35 |  |  |
|  | *ndhA* | SSC | - | 123910 | 126141 | 558 | 1137 | 537 |  |  |
|  | *trnA-UGC* | IRb | - | 138557 | 139431 | 35 | 802 | 38 |  |  |
|  | *trnI-GAU* | IRb | - | 139504 | 140533 | 30 | 958 | 42 |  |  |
|  | *rps12-3' end* | IRb | + | 144271 | 145068 | 232 | 540 | 26 |  |  |
|  | *ndhB* | IRb | + | 145918 | 148136 | 777 | 686 | 756 |  |  |
|  | *ycf15-orf* | IRb | - | 149109 | 149641 | 90 | 284 | 159 |  |  |
|  | *rpl2* | IRb | + | 157204 | 158693 | 393 | 626 | 471 |  |  |
| *S. siamensis* | *trnK-UUU* | LSC | - | 1629 | 4209 | 37 | 2509 | 35 |  |  |
|  | *rps16* | LSC | - | 5199 | 6308 | 48 | 849 | 213 |  |  |
|  | *atpF* | LSC | - | 13349 | 14652 | 147 | 749 | 408 |  |  |
|  | *rpoC1* | LSC | - | 22633 | 25421 | 435 | 743 | 1611 |  |  |
|  | *ycf3* | LSC | - | 45569 | 47546 | 129 | 729 | 225 | 742 | 153 |
|  | *trnL-UAA* | LSC | + | 50611 | 51226 | 35 | 531 | 50 |  |  |
|  | *trnV-UAC* | LSC | - | 54913 | 55597 | 38 | 610 | 37 |  |  |
|  | *clpP* | LSC | - | 73635 | 75681 | 69 | 835 | 297 | 621 | 225 |
|  | *petB* | LSC | + | 78619 | 80052 | 6 | 786 | 642 |  |  |
|  | *petD* | LSC | + | 80245 | 81450 | 8 | 723 | 475 |  |  |
|  | *rpl2* | IRa | - | 88135 | 89624 | 393 | 626 | 471 |  |  |
|  | *ycf15-orf* | IRa | + | 97187 | 97719 | 159 | 284 | 90 |  |  |
|  | *ndhB* | IRa | - | 98692 | 100910 | 777 | 686 | 756 |  |  |
|  | *rps12-5' end* | IRa | - | 101760 | 102557 | 232 | 540 | 26 |  |  |
|  | *trnI-GAU* | IRa | + | 106295 | 107324 | 42 | 958 | 30 |  |  |
|  | *trnA-UGC* | IRa | + | 107397 | 108271 | 38 | 802 | 35 |  |  |
|  | *ndhA* | SSC | - | 124342 | 126573 | 558 | 1137 | 537 |  |  |
|  | *trnA-UGC* | IRb | - | 138989 | 139863 | 38 | 802 | 35 |  |  |
|  | *trnI-GAU* | IRb | - | 139936 | 140965 | 42 | 958 | 30 |  |  |
|  | *rps12-3' end* | IRb | + | 144703 | 145500 | 232 | 540 | 26 |  |  |
|  | *ndhB* | IRb | + | 146350 | 148568 | 777 | 686 | 756 |  |  |
|  | *ycf15-orf* | IRb | - | 149541 | 150073 | 159 | 284 | 90 |  |  |
|  | *rpl2* | IRb | + | 157636 | 159125 | 393 | 626 | 471 |  |  |
