# Supplementary material for: Complete chloroplast genomes of two Siraitia Merrill species: Comparative analysis, positive selection and novel molecular marker development
Source: PLoS One. 2019 Dec 20;14(12):e0226865. doi: 10.1371/journal.pone.0226865 (PMC6924677; doi:10.1371/journal.pone.0226865)
Supplement: S6 Table — (DOCX) [file pone.0226865.s009.docx]

**S6 Table. Comparisons among the chloroplast genome characteristics of *S. grosvenorii* and *S. siamensis*, and other six Cucurbitaceae species.**

| **Species** | ***Siraitia grosvenorii*** | ***Siraitia siamensis*** | ***Momordica Charantia*** | ***Citrullus lanatus*** | ***Cucumis sativus*** | ***Lagenaria siceraria*** | ***Trichosanthes kirilowii*** | ***Gynostemma laxiflorum*** |
| --- | --- | --- | --- | --- | --- | --- | --- | --- |
| Accession No. | MK755853 | MK755854 | MG022622 | KY014105 | AJ970307 | MG022623 | MK036046 | MF136486 |
| Genome size (bp) | 158,757 | 159,190 | 158,844 | 156,906 | 155,293 | 157,145 | 157,481 | 158,273 |
| LSC length (bp) | 87,625 | 88069 | 88,374 | 86,845 | 86,688 | 86,843 | 86,478 | 87,047 |
| SSC Length (bp) | 18,556 | 18543 | 18,006 | 17,897 | 18,223 | 18,008 | 18,467 | 18,708 |
| IR Length (bp) | 26,288 | 26289 | 26,232 | 26,082 | 25,193 | 26,147 | 26,268 | 26259 |
| Overall GC content (%) | 36.9 | 36.9 | 36.7 | 37.2 | 37.1 | 37.1 | 37.1 | 37.0 |
| Number of genes | 134 | 134 | 130 | 122 | 132 | 130 | 129 | 132 |
| Number of protein-coding genes | 89 | 89 | 84 | 85 | 86 | 85 | 85 | 87 |
| Number of tRNAs | 37 | 37 | 38 | 29 | 38 | 37 | 36 | 37 |
| Number of rRNAs | 8 | 8 | 8 | 8 | 8 | 8 | 8 | 8 |
