# Supplementary material for: Complete chloroplast genomes of two Siraitia Merrill species: Comparative analysis, positive selection and novel molecular marker development
Source: PLoS One. 2019 Dec 20;14(12):e0226865. doi: 10.1371/journal.pone.0226865 (PMC6924677; doi:10.1371/journal.pone.0226865)
Supplement: S7 Table — (DOCX) [file pone.0226865.s010.docx]

**S7 Table. Codon usage and codon-anticodon recognition in all protein-coding genes of the chloroplast genomes of two *Siraitia* species.**

| **Amino acid** | **Codon** | **tRNA** | ***Siraitia grosvenorii*** | | | | ***Siraitia siamensis*** | | | |
| --- | --- | --- | --- | --- | --- | --- | --- | --- | --- | --- |
|  |  |  | **RSCU** | **Number** | **Fraction** | **Frequency** | **RSCU** | **Number** | **Fraction** | **Frequency** |
| Ala | GCA | *trnA-UGC* | 1.09 | 379 | 0.273 | 14.297 | 1.09 | 381 | 0.274 | 14.373 |
| Ala | GCC | *-* | 0.65 | 227 | 0.163 | 8.563 | 0.67 | 232 | 0.167 | 8.752 |
| Ala | GCG | *-* | 0.42 | 146 | 0.105 | 5.508 | 0.42 | 145 | 0.104 | 5.470 |
| Ala | GCU | *-* | 1.84 | 638 | 0.459 | 24.067 | 1.82 | 635 | 0.456 | 23.955 |
| Cys | UGC | *trnC-GCA* | 0.53 | 80 | 0.267 | 3.018 | 0.53 | 80 | 0.266 | 3.018 |
| Cys | UGU | *-* | 1.47 | 220 | 0.733 | 8.299 | 1.47 | 221 | 0.734 | 8.337 |
| Asp | GAC | *trnD-GUC* | 0.37 | 205 | 0.187 | 7.733 | 0.38 | 209 | 0.191 | 7.884 |
| Asp | GAU | *-* | 1.63 | 890 | 0.813 | 33.574 | 1.62 | 887 | 0.809 | 33.462 |
| Glu | GAA | *trnE-UUC* | 1.49 | 1045 | 0.745 | 39.421 | 1.49 | 1046 | 0.744 | 39.460 |
| Glu | GAG | *-* | 0.51 | 357 | 0.255 | 13.467 | 0.51 | 359 | 0.256 | 13.543 |
| Phe | UUC | *trnF-GAA* | 0.71 | 537 | 0.357 | 20.257 | 0.71 | 534 | 0.356 | 20.145 |
| Phe | UUU | *-* | 1.29 | 966 | 0.643 | 36.44 | 1.29 | 967 | 0.644 | 36.480 |
| Gly | GGA | *trnG-UCC* | 1.63 | 733 | 0.406 | 27.651 | 1.63 | 734 | 0.407 | 27.690 |
| Gly | GGC | *-* | 0.39 | 177 | 0.098 | 6.677 | 0.38 | 173 | 0.096 | 6.526 |
| Gly | GGG | *-* | 0.65 | 291 | 0.161 | 10.977 | 0.64 | 290 | 0.161 | 10.940 |
| Gly | GGU | *-* | 1.34 | 603 | 0.334 | 22.747 | 1.34 | 605 | 0.336 | 22.823 |
| His | CAC | *trnH-GUG* | 0.47 | 149 | 0.235 | 5.621 | 0.47 | 149 | 0.234 | 5.621 |
| His | CAU | *-* | 1.53 | 486 | 0.765 | 18.333 | 1.53 | 487 | 0.766 | 18.372 |
| Ile | AUA | *trnI-CAU* | 0.93 | 703 | 0.308 | 26.519 | 0.92 | 701 | 0.307 | 26.445 |
| Ile | AUC | *trnI-GAU* | 0.62 | 471 | 0.207 | 17.768 | 0.62 | 472 | 0.207 | 17.806 |
| Ile | AUU | *-* | 1.45 | 1105 | 0.485 | 41.684 | 1.46 | 1107 | 0.486 | 41.761 |
| Lys | AAA | *trnK-UUU* | 1.50 | 1053 | 0.751 | 39.722 | 1.50 | 1051 | 0.751 | 39.648 |
| Lys | AAG | *-* | 0.50 | 349 | 0.249 | 13.165 | 0.50 | 349 | 0.249 | 13.166 |
| Leu | CUA | *trnL-UAG* | 0.84 | 388 | 0.140 | 14.637 | 0.84 | 390 | 0.140 | 14.713 |
| Leu | CUC | *-* | 0.43 | 198 | 0.071 | 7.469 | 0.42 | 196 | 0.071 | 7.394 |
| Leu | CUG | *-* | 0.41 | 190 | 0.068 | 7.167 | 0.41 | 190 | 0.068 | 7.168 |
| Leu | CUU | *-* | 1.25 | 579 | 0.208 | 21.842 | 1.25 | 577 | 0.208 | 21.767 |
| Leu | UUA | *trnL-UAA* | 1.87 | 867 | 0.312 | 32.706 | 1.87 | 866 | 0.312 | 32.669 |
| Leu | UUG | *trnL-CAA* | 1.20 | 555 | 0.200 | 20.936 | 1.21 | 560 | 0.202 | 21.126 |
| Met | AUG | *trn(f)M-CAU* | 1.00 | 623 | 1.000 | 23.501 | 1.00 | 623 | 1.000 | 23.502 |
| Asn | AAC | *trnN-GUU* | 0.46 | 292 | 0.228 | 11.015 | 0.46 | 292 | 0.228 | 11.016 |
| Asn | AAU | *-* | 1.54 | 986 | 0.772 | 37.195 | 1.54 | 988 | 0.772 | 37.272 |
| Pro | CCA | *trnP-UGG* | 1.16 | 319 | 0.291 | 12.034 | 1.17 | 320 | 0.292 | 12.072 |
| Pro | CCC | *trnP-GGG* | 0.75 | 206 | 0.188 | 7.771 | 0.75 | 205 | 0.187 | 7.734 |
| Pro | CCG | *-* | 0.54 | 148 | 0.135 | 5.583 | 0.54 | 148 | 0.135 | 5.583 |
| Pro | CCU | *-* | 1.54 | 423 | 0.386 | 15.957 | 1.55 | 424 | 0.387 | 15.995 |
| Gln | CAA | *trnQ-UUG* | 1.54 | 727 | 0.772 | 27.425 | 1.55 | 722 | 0.773 | 27.237 |
| Gln | CAG | *-* | 0.46 | 215 | 0.228 | 8.11 | 0.45 | 212 | 0.227 | 7.998 |
| Arg | AGA | *trnR-UCU* | 1.84 | 490 | 0.306 | 18.484 | 1.83 | 489 | 0.305 | 18.447 |
| Arg | AGG | *-* | 0.65 | 173 | 0.108 | 6.526 | 0.64 | 171 | 0.107 | 6.451 |
| Arg | CGA | *-* | 1.41 | 375 | 0.234 | 14.146 | 1.40 | 374 | 0.233 | 14.109 |
| Arg | CGC | *-* | 0.39 | 105 | 0.066 | 3.961 | 0.40 | 106 | 0.066 | 3.999 |
| Arg | CGG | *-* | 0.40 | 106 | 0.066 | 3.999 | 0.41 | 110 | 0.069 | 4.150 |
| Arg | CGU | *trnR-ACG* | 1.32 | 352 | 0.220 | 13.279 | 1.32 | 352 | 0.220 | 13.279 |
| Ser | AGC | *trnS-GCU* | 0.38 | 128 | 0.063 | 4.829 | 0.38 | 131 | 0.064 | 4.942 |
| Ser | AGU | *-* | 1.17 | 398 | 0.194 | 15.014 | 1.16 | 397 | 0.194 | 14.977 |
| Ser | UCA | *trnS-UGA* | 1.27 | 433 | 0.212 | 16.334 | 1.27 | 434 | 0.212 | 16.372 |

**S7 Table.** Cont.

| **Amino acid** | **Codon** | **tRNA** | ***Siraitia grosvenorii*** | | | | ***Siraitia siamensis*** | | | |
| --- | --- | --- | --- | --- | --- | --- | --- | --- | --- | --- |
|  |  |  | **RSCU** | **Number** | **Fraction** | **Frequency** | **RSCU** | **Number** | **Fraction** | **Frequency** |
| Ser | UCC | *trnS-GGA* | 0.98 | 334 | 0.163 | 12.599 | 0.98 | 334 | 0.163 | 12.600 |
| Ser | UCG | *-* | 0.56 | 190 | 0.093 | 7.167 | 0.56 | 191 | 0.093 | 7.205 |
| Ser | UCU | *-* | 1.65 | 564 | 0.276 | 21.276 | 1.65 | 562 | 0.274 | 21.201 |
| Thr | ACA | *trnT-UGU* | 1.21 | 404 | 0.302 | 15.240 | 1.20 | 401 | 0.300 | 15.128 |
| Thr | ACC | *trnT-GGU* | 0.74 | 248 | 0.185 | 9.355 | 0.74 | 247 | 0.185 | 9.318 |
| Thr | ACG | *-* | 0.43 | 145 | 0.108 | 5.470 | 0.44 | 146 | 0.109 | 5.508 |
| Thr | ACU | *-* | 1.62 | 540 | 0.404 | 20.370 | 1.62 | 542 | 0.406 | 20.447 |
| Val | GUA | *trnV-UAC* | 1.50 | 540 | 0.375 | 20.370 | 1.50 | 539 | 0.375 | 20.333 |
| Val | GUC | *trnV-GAC* | 0.51 | 183 | 0.127 | 6.903 | 0.51 | 184 | 0.128 | 6.941 |
| Val | GUG | *-* | 0.55 | 199 | 0.138 | 7.507 | 0.55 | 199 | 0.138 | 7.507 |
| Val | GUU | *-* | 1.44 | 519 | 0.360 | 19.578 | 1.44 | 517 | 0.359 | 19.504 |
| Trp | UGG | *trnW-CCA* | 1.00 | 471 | 1.000 | 17.768 | 1.00 | 470 | 1.000 | 17.730 |
| Tyr | UAC | *trnY-GUA* | 0.39 | 195 | 0.196 | 7.356 | 0.39 | 195 | 0.196 | 7.356 |
| Tyr | UAU | *-* | 1.61 | 802 | 0.804 | 30.254 | 1.61 | 801 | 0.804 | 30.217 |
| Stop | UAA | *-* | 1.89 | 56 | 0.629 | 2.112 | 1.89 | 56 | 0.629 | 2.113 |
| Stop | UAG | *-* | 0.51 | 15 | 0.169 | 0.566 | 0.51 | 15 | 0.169 | 0.566 |
| Stop | UGA | *-* | 0.61 | 18 | 0.202 | 0.679 | 0.61 | 18 | 0.202 | 0.679 |
