# Supplementary material for: Complete chloroplast genomes of two Siraitia Merrill species: Comparative analysis, positive selection and novel molecular marker development
Source: PLoS One. 2019 Dec 20;14(12):e0226865. doi: 10.1371/journal.pone.0226865 (PMC6924677; doi:10.1371/journal.pone.0226865)
Supplement: S8 Table — (DOCX) [file pone.0226865.s011.docx]

**S8 Table. Types and amounts of SSRs in the *S. grosvenorii* and *S. siamensis* chloroplast genomes.**

| **SSR Type** | **Repeat Unit** | ***S. grosvenorii*** | | ***S. siamensis*** | |
| --- | --- | --- | --- | --- | --- |
|  |  | **Amount** | **Ratio (%)** | **Amount** | **Ratio (%)** |
| Mono | A/T | 173 | 97.2 | 175 | 97.8 |
|  | C/G | 5 | 2.8 | 4 | 2.2 |
| Di | AC/GT | 2 | 3.5 | 2 | 3.6 |
|  | AG/CT | 15 | 26.3 | 15 | 26.8 |
|  | AT/TA | 39 | 68.4 | 38 | 67.8 |
|  | CG/CG | 1 | 1.8 | 1 | 1.8 |
| Tri | AAT/ATT | 6 | 100 | 7 | 100 |
| Tetra | AAAC/GTTT | 1 | 10 | 1 | 11.1 |
|  | AAAT/ATTT | 6 | 60 | 7 | 77.8 |
|  | AAAG/CTTT | 1 | 10 | 0 | 0 |
|  | AAGT/ACTT | 1 | 10 | 1 | 11.1 |
|  | AATG/ATTC | 1 | 10 | 0 | 0 |
| Penta | AAAAG/CTTTT | 1 | 100 | 0 | 0 |
|  | AAGTC/ACTTG | 0 | 0 | 1 | 100 |
| Hexa | AAAAAT/ATTTTT | 0 | 0 | 1 | 100 |
